# HIV Testing among Outpatients with Medicaid and Commercial Insurance

**DOI:** 10.1371/journal.pone.0144965

**Published:** 2015-12-14

**Authors:** Patricia M. Dietz, Michelle Van Handel, Huisheng Wang, Philip J. Peters, Jun Zhang, Abigail Viall, Bernard M. Branson

**Affiliations:** Division of HIV/AIDS Prevention, Centers for Disease Control and Prevention, Atlanta, Georgia, United States of America; Leibniz Institute for Prevention Research and Epidemiology (BIPS), GERMANY

## Abstract

**Objective:**

To assess HIV testing and factors associated with receipt of testing among persons with Medicaid and commercial insurance during 2012.

**Methods:**

Outpatient and laboratory claims were analyzed from two databases: all Medicaid claims from six states and all claims from Medicaid health plans from four other states and a large national convenience sample of patients with commercial insurance in the United States. We excluded those aged <13 years and >64 years, enrolled <9 of the 12 months, pregnant females, and previously diagnosed with HIV. We identified patients with new HIV diagnoses that followed (did not precede) the HIV test, using HIV ICD-9 codes. HIV testing percentages were assessed by patient demographics and other tests or diagnoses that occurred during the same visit.

**Results:**

During 2012, 89,242 of 2,069,536 patients (4.3%) with Medicaid had at least one HIV test, and 850 (1.0%) of those tested received a new HIV diagnosis. Among 27,206,804 patients with commercial insurance, 757,646 (2.8%) had at least one HIV test, and 5,884 (0.8%) of those tested received a new HIV diagnosis. During visits that included an HIV test, 80.2% of Medicaid and 83.0% of commercial insurance claims also included a test or diagnosis for a sexually transmitted infection (STI), and/or Hepatitis B or C virus at the same visit.

**Conclusions:**

HIV testing primarily took place concurrently with screening or diagnoses for STIs or Hepatitis B or C. We found little evidence to suggest routine screening for HIV infection was widespread.

## Introduction

Approximately 13% of the estimated 1 million persons living with HIV in the United States are unaware of their infection and unable to benefit from effective treatment that improves health and reduces transmission risk [1]. HIV testing can be targeted towards individuals with behaviors that increase their risk of HIV acquisition and transmission or can be routine, meaning that all persons receiving care in a health care setting are tested at least once regardless of risk [2]. In health care settings with ≥0.1% undiagnosed HIV prevalence, routine HIV testing is cost-effective [3]. Since 2006, the Centers for Disease Control and Prevention (CDC) has recommended that providers routinely screen adolescents and adults for HIV, and perform testing at least annually for individuals who engage in behaviors that increase their risk of acquiring and transmitting HIV, and in 2013, the U.S. Preventive Services Task Force (USPSTF) issued similar recommendations [4, 5]. CDC monitors the percentage of persons who are tested for HIV to better understand the uptake of testing recommendations and to effectively deploy HIV testing resources.

Data from the National Health Interview Survey have been used to provide annual estimates of self-reported HIV testing [6]. In 2010, an estimated 45% of persons in the U.S. aged 18–64 years had ever been tested for HIV and 10.1% reported being tested within the preceding 12 months [6]. Limitations of these estimates include potential bias from self-reported testing [7, 8], and the inability to assess if the testing was routine, initiated due to symptoms, or part of targeted testing outside the health care system. Differentiating between routine and targeted testing in health care settings could inform evaluation of the uptake of the HIV screening recommendations.

Administrative claims data can provide information on other diagnoses and testing performed during the same visit as the HIV test, allowing assessment of co-morbid conditions and concurrent tests for other infections. We sought to estimate the yearly percentage of HIV tests among persons with Medicaid and commercial insurance to better understand HIV testing patterns in these populations, using administrative claims data. We also assessed whether the HIV test might be part of a routine testing protocol or stimulated by symptoms or reported risk behaviors by examining testing for other sexually transmitted infections (STI), and hepatitis B or hepatitis C virus (HBV or HCV) infections conducted during the same visit as the HIV test.

## Methods

**Fig 1:**
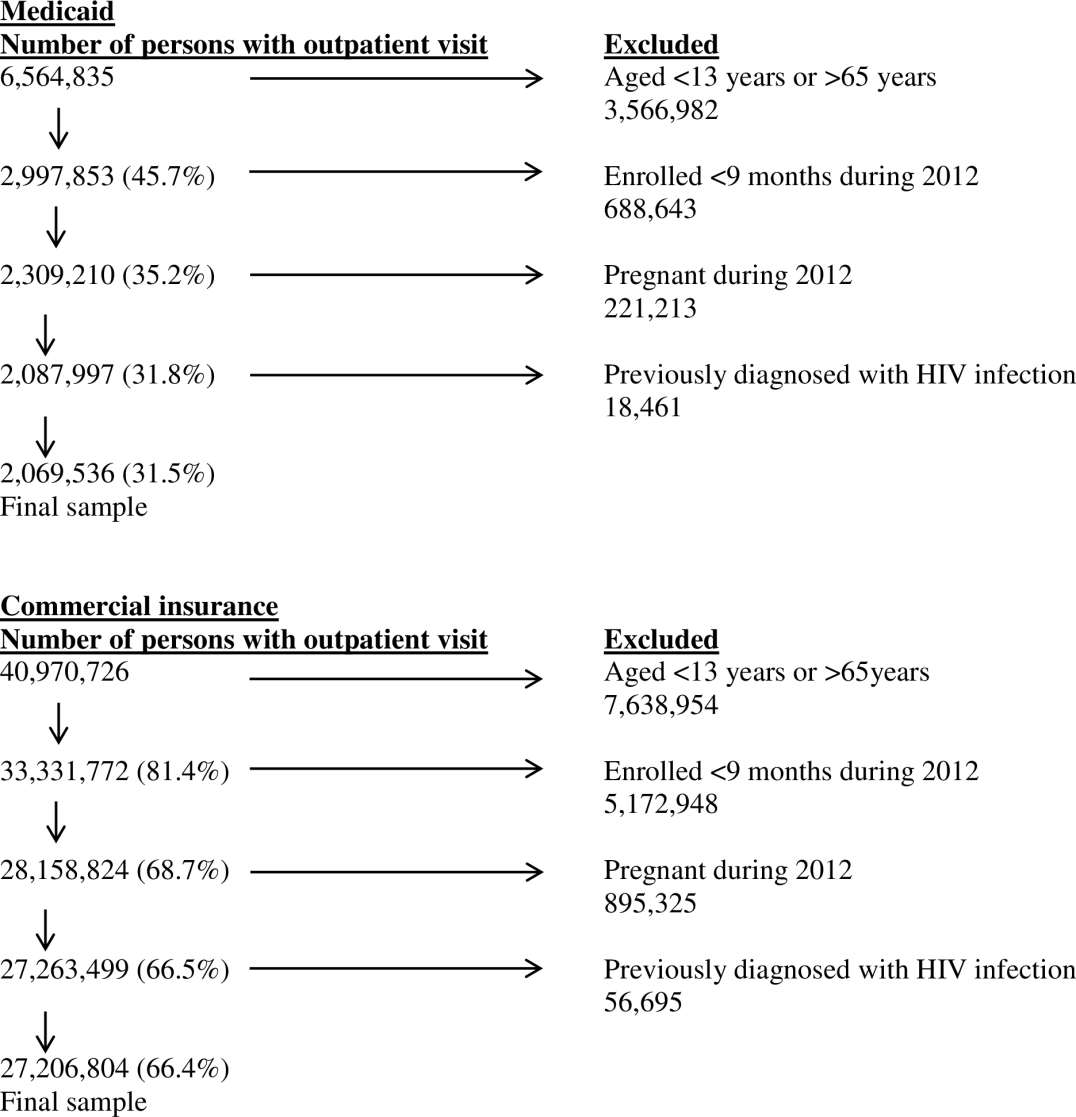
Number of persons exclude and final sample, MarketScan 2012 (A). Eligible persons with Medicaid. (B) Eligible patients with commercial insurance.

We analyzed 2012 Truven Health MarketScan Multi-State Medicaid and Commercial Insurance Databases (http://truvenhealth.com/your-healthcare-focus/life-sciences/data-databases-and-online-tools, accessed 9/19/2014). The Medicaid database included Medicaid outpatient claims data for 6,564,835 patients from six geographically diverse states and from four Medicaid health plans in four other states. State identification was not included in the Medicaid data as requested by states in their confidentiality agreements with Truven. The commercial insurance database included a convenience sample of outpatient claims data for 40,970,726 patients from all states, representing approximately 25% of all persons insured with commercial insurance in the United States in 2012. The eligibility criteria for both study samples included an age of 13 to 64 years as of January 1, 2012; enrollment for at least 9 of the 12 months during 2012; not pregnant during 2012; and no evidence of a previous HIV diagnosis in 2012 or 2011. We restricted the age group to the ages recommended to be screened for HIV and excluded pregnant women because screening for HIV is well documented in this population [4, 5]. Persons who had an HIV diagnosis in 2012 before the date of the first HIV test were considered previously diagnosed. In addition, the 2011 MarketScan Medicaid or commercial insurance databases were reviewed and persons with an HIV diagnosis code in 2011 were excluded from this analysis to remove any person with a previous HIV diagnosis from the sample (Fig 1A and Fig 1B). The final analysis samples comprised 2,069,536 eligible patients with Medicaid (31.5% of the total) and 27,206,804 eligible patients with commercial insurance (66.4% of the total).

We restricted our analysis to claims from outpatient visits, including laboratory claims and emergency room visits, because review of inpatient claims found very few HIV tests. We assessed the percentage of patients who had been tested at least once for HIV during 2012 stratified by patient characteristics and same day testing for or diagnosis of STI, and HBV or HCV infections. Chlamydia and gonorrhea tests among females aged 13 to 24 years during visits for preventive care were not counted as tests for STIs because these likely represented routine screening [9]; in our analysis, tests for STIs were a proxy for HIV testing performed because of patient symptoms or reported risk behaviors. In August 2012, CDC published recommendations for routine HCV screening for persons born between 1945 and 1965 [10]. We did not exclude HCV tests because the recommendations were released 8 months into the study year and we thought that it was unlikely that the new recommendations would be immediately incorporated into clinical practice.

The International Classification of Diseases, Clinical Modification (ICD-9-CM) codes and Current Procedural Terminology (CPT) codes were used to identify tests or diagnoses for HIV infection, other STIs, HBV, and HCV (Table 1). We calculated the percentage of patients with a new HIV diagnosis by dividing the number of patients with an HIV diagnosis by the number of patients with at least one HIV test during the year. We stratified all analyses by sex because previous studies have documented higher percentages of HIV testing among females [6]. Chi square tests were used to assess the statistical significance (p<0.05) of differences in the distribution of HIV testing by patient characteristics. Logistic regression models estimated the independent associations of age and race/ethnicity characteristics of persons receiving an HIV test and an HIV diagnosis. These models were run separately for males and females and they were estimated among Medicaid patients but not among commercially insure patients since race/ethnicity was not available for commercially insured patients. We used SAS 9.3 (SAS Institute, Cary, NC) for all analyses [11]. The large sample size of the Medicaid (2,069,536 persons) and commercially insured (27,206,804 persons) databases allowed sufficient power to detect any clinically relevant differences.

**Table 1:** International Classification of Diseases, Clinical Modification (ICD-9-CM) codes and Current Procedural Terminology (CPT) codes for tests and diagnoses for HIV, sexually transmitted infections, and Hepatitis B or C virus, 2012.

Parameter	ICD-9-CM codes	CPT codes
HIV diagnosis	042, 079.53, 795.71, V08	
HIV test		86689, 86701, 86703, 87389, 87390, 87534,
		87535
Syphilis	091, 091.1, 091.2, 091.3, 091.4, 091.69, 091.7,	
	091.89, 091.9	
Syphilis tests		87164, 86781, 86780, 86592, 86593
Chlamydia or gonorrhea infection	099.41, 099.5, 099.51, 099.52, 099.53,	
	099.54,099.55, 099.56, 099.59, V73.98, 098.xx	
Chlamydia or gonorrhea tests/diagnoses	V74.5, V73.88, V73.89, 078.88, 079.88, 079.98	86631, 86632, 87110, 87270, 87320, 87490-
		87492, 87590–87592, 87810, 87850
Genital herpes diagnosis	054.1X	
Herpes simplex tests		87273, 87274, 87528, 87529, 87530, 86696,
		87207
Genital wart diagnosis	078.1, 078.11, 078.19	
Pelvic inflammatory disease	614.0, 614.3, 614.5, 615.0	
Other nongonococcal urethritis diagnoses	099.4, 099.49	
Chancroid diagnosis	099.0	
Granuloma inguinale diagnosis	099.2	
Lymphogranuloma venereum	099.1	86729
Human papillomavirus	079.4, 795.05, 795.09	87620, 87621, 87622
STD exposure, counseling, screening	V01.6, V65.45, V74.5	
Hepatitis B virus	070.20, 070.21,070.30, 070.31	80074, 86704–86707, 87340, 87341, 87350,
		87515, 87516, 87517
Hepatitis C virus	070.51	80074, 86803, 86804, 87520–87522,
Trichomoniasis	131.xx	87808, 87810, 87850, 87660
Well visit		99384–99386, 99394–99396, HCPCS Code
		G0402, V70.0
Well visit with possible medical compliant		99203–99205, 99213–99215
Well visits with specialty care		99243, 99244, 99245

## Results

Table 2 lists the demographic characteristics and HIV testing percentages of patients with Medicaid and commercial insurance. More than 44% of patients with Medicaid were aged 13–19 years, compared with 12% of patients with commercial insurance. Approximately half of patients with Medicaid were white (47.4%) and 35.3% were black/African American. Overall, 4.3% of patients with Medicaid and 2.8% of patients with commercial insurance had at least one test for HIV in 2012, p<0.001. Very few had more than one HIV test: 0.4% of patients with Medicaid and 0.2% of patients with commercial insurance. A higher percentage of females than males were tested for HIV in both samples but the magnitude of the difference was more pronounced in the Medicaid sample (5.7% versus 2.4%) than in the commercial insurance sample (3.0% versus 2.5%), p<0.001 for both samples. Among Medicaid patients, of those tested, 850 (1.0%) received a diagnosis of HIV, and among patients with commercial insurance, 5,884 (0.8%) received a diagnosis of HIV. Overall, 14.1% of patients with Medicaid and 13.3% of patients with commercial insurance were tested for at least one STI/HBV/HCV during 2012. Of those tested, 35,872 (12.3%) of Medicaid patients and 281,063 (7.7%) of patients with commercial insurance received a diagnosis for at least one STI/HBV/HCV. Co-infection with a STI/ HBV/HCV at any time during 2012 occurred approximately in one in five of those with an HIV diagnosis: 18.4% of Medicaid patients and 18.9% of patients with commercial insurance.

**Table 2:** Demographics and HIV testing by type of health plan, Medicaid and commercial insurance, 2012.

	Medicaid	Commercial insurance
	N = 2,069,536	N = 27,206,804
	%	%
**Age (yrs)**		
13–14	15.7	3.3
15–19	28.5	9.4
20–29	13.2	14.1
30–39	12.7	16.4
40–49	11.5	22.3
50–64	18.4	34.5
**Sex**		
Female	58.2	53.9
Male	41.8	46.1
**Race/ethnicity**		
Non-Hispanic white	47.4	NA
Non-Hispanic black	35.3	NA
Hispanic/Latino	4.1	NA
Non-Hispanic other	13.2	NA
**HIV testing frequency**		
0	95.7	97.2
At least 1 test	4.3	2.8
1	3.9	2.6
>1	0.4	0.2
Tested positive among tested^a^	1.0	0.8
**STI/HBV/HCV testing frequency**		
None	85.9	86.7
Any	14.1	13.3
Tested positive among tested^b^	12.3	7.7
**Co-infection with STI/HBV/HCV among those with HIV diagnosis** ^c^	18.4	18.9
**Co-infection with HIV among those with STI/HBV/HCV diagnosis** ^**d**^	1.0	1.5

STI Sexually Transmitted Infection; HBV Hepatitis B virus; HCV Hepatitis C virus

^a^ Percentage positive is among those tested (n = 850/89,242 for Medicaid and n = 5,884/757,646 for commercially insured) at any time during the year.

^b^ Percentage positive is among those tested for STI/HBV/HCV for Medicaid (n = 35,872/291,982) and for commercially insured (n = 281,063/3,632,566).

^c^ Percentage is among those with HIV diagnoses and also tested for a STI/HBV/HCV during the year for Medicaid (n = 139/754) and for commercially insured (n = 978/5,181)

^d^ Percentage is among those with STI/Hepatitis B or C diagnoses for Medicaid (139/13,505) and for commercially insured (n = 978/64,957). NA: not available.

### HIV Testing

A higher percentage of females with Medicaid received HIV testing compared with females with commercial insurance (5.7% versus 3.0%, p<0.0001). For females with either Medicaid or commercial insurance, a greater percentage of those aged 20–29 years had been tested for HIV (10.4% and 7.3%, respectively) than those in younger or older age groups (range 1.2%-8.0% Medicaid, 0.5%-5.1% commercial insurance), p<0.0001 for both samples (Table 3). Among females with Medicaid, blacks/African Americans were more likely to have been tested (8.5%) than whites (4.0%), Hispanics/Latinas (4.0%), and females of other races/ethnicities (4.4%), p<0.0001 (Table 3).

**Table 3:** HIV tests by demographic characteristics and type of healthcare coverage among females and males.

	Medicaid		Commercial insurance	
	% Females Tested^a^	% Males Tested^a^	% Females Tested^a^	% Males Tested^a^
	(N = 68,469/	(N = 20,773/	(N = 444,362/	(N = 313,284/
	1,204,341)	865,195)	14,660,376)	12,546,428)
**Total**	**5.7**	**2.4**	**3.0**	**2.5**
**Age (yrs)**				
13–14	1.2	0.5	0.5	0.1
15–19	6.6	2.9	2.7	1.4
20–29	10.4	3.7	7.3	5.3
30–39	8.0	3.0	5.1	4.0
40–49	4.8	2.7	2.5	2.4
50–64	2.1	2.4	1.0	1.3
**Race/ethnicity**					
White	4.0	1.6	NA	NA
Black	8.5	3.7	NA	NA
Hispanic/Latino	4.0	1.5	NA	NA
Other	4.4	2.2	NA	NA

NA = not available.

All chi square tests for specific characteristics (e.g., age) and % tested, and for specific characteristics (e.g., age) and % diagnosed had p<0.0001.

^a^% tested defined as number of persons tested / number of persons in the sample.

Among female Medicaid patients, after adjusting for race/ethnicity, females aged 15–19 years (odds ratio (OR) 3.1, 95% confidence interval (CI) 3.0, 3.2), aged 20–29 years (OR = 5.1, 95% CI 5.0, 5.3), aged 30–39 years (OR = 3.9, 95% CI 3.8, 4.1), and aged 40–49 years (OR = 2.3, 95% CI 2.2, 2.4) had greater odds of receiving an HIV test compared with females aged 50–64 years. After adjusting for age, black/African Americans females (OR = 2.2, 95% CI 2.2, 2.3), Hispanic/Latina females (OR = 1.1, 95% CI 1.1, 1.2), and females of other race/ethnicity (OR = 1.1, 95% CI 1.1, 1.1), had greater odds of receiving an HIV test compared with white females (data not shown).

A slightly lower percentage of males with Medicaid than males with commercial insurance were tested for HIV (2.4% versus 2.5%, p<0.0001). A greater percentage of males aged 20–29 years had been tested for HIV (3.7% for Medicaid and 5.3% for commercial insurance) than those in younger or older age groups (range 0.5%-3.0% for Medicaid, 0.1%-4.0% for commercial insurance), p<0.001 for both samples (Table 3). The percentage tested for HIV was highest among non-Hispanic blacks/African Americans (3.7%) compared with non-Hispanic whites (1.6%), Hispanics/Latinos (1.5%), and non-Hispanic males of other race/ethnicity (2.2%), p<0.0001, among those enrolled in Medicaid.

Among male Medicaid patients, after adjusting for race/ethnicity, males aged 15–19 years (OR = 1.2, 95% CI 1.1, 1.2), males aged 20–29 years (OR = 1.6, 95% CI 1.5, 1.7), aged 30–39 years (OR = 1.3, 95% CI 1.2, 1.4), and aged 40–49 years (OR = 1.2, 95% CI 1.1, 1.3) had greater odds of receiving an HIV test compared with males aged 50–64 years. After adjusting for age, black/African Americans males (OR = 2.5, 95% CI 2.4, 2.6), Hispanic/Latino males (OR = 1.1, 95% CI 1.1, 1.2), and males of other race/ethnicity (OR = 1.4, 95% CI 1.3, 1.5), had greater odds of receiving an HIV test compared with white males (data not shown).

### New HIV Diagnoses among Those Tested

The percentage of females with a new HIV diagnosis was higher for females with Medicaid (0.9%) compared with females with commercial insurance (0.5%), p<0.0001. The highest percentage of females who tested positive for HIV were among those aged 50–64 years (1.3% Medicaid, 0.7% commercial insurance), and lowest among those aged 13–14 years (0.4% Medicaid, 0.2% commercial insurance), p<0.0001 for both samples. Among females with Medicaid, the percentage with a new HIV diagnosis was highest among blacks/African Americans (1.0%) compared with whites (0.7%), Hispanics/Latinas (0.5%), and females of other race/ethnicity (0.7%), p<0.0001 (Table 4).

**Table 4:** HIV diagnoses by demographic characteristics and type of healthcare coverage among females and males.

	Medicaid		Commercial insurance	
	% Females	% Males	% Females	% Males
	diagnosed^a^	diagnosed^a^	diagnosed^a^	diagnosed^a^
	(N = 586/	(N = 264/	(N = 2,078/	(N = 3,806/
	68,469)	20,773)	444,362)	313,284)
**Total**	**0.9**	**1.3**	**0.5**	**1.2**
**Age (yrs)**				
13–14	0.4	0.3	0.2	0.6
15–19	0.5	0.7	0.4	0.7
20–29	0.9	1.5	0.4	1.1
30–39	1.0	1.8	0.5	1.1
40–49	1.2	2.4	0.6	1.4
50–64	1.3	1.6	0.7	1.3
**Race/ethnicity**				
White	0.7	0.8	NA	NA
Black	1.0	1.6	NA	NA
Hispanic/Latino	0.5	1.1	NA	NA
Other	0.7	1.2	NA	NA

NA = not available.

All chi square tests for specific characteristics (e.g., age) and % tested, and for specific characteristics (e.g., age) and % diagnosed had p<0.0001.

^a^% diagnosed defined as the number of persons diagnosed with HIV infection / number of persons tested for HIV.

Among female Medicaid patients, after adjusting for race/ethnicity, compared with females aged 50–64 years, females aged 13–14 years (OR = 0.2, 95% CI 0.1, 0.3) had lower odds of having HIV diagnosed, and females aged 20–29 years (OR = 3.2, 95% CI 2.4, 4.3), aged 30–39 years (OR = 2.9, 95% CI 2.1, 3.9), and aged 40–49 years (OR = 2.1, 95% CI 1.5, 3.0) had higher odds. After adjusting for age, black/African Americans females (OR = 3.3, 95% CI 2.7, 4.0) had higher odds of having HIV diagnosed compared with white females, and Hispanic/Latina and females of other race/ethnicity had similar odds (data not shown).

The percentage of males who were diagnosed with HIV infection was 1.3% of those tested with Medicaid and 1.2% of those tested with commercial insurance, p<0.0001. The percentage of males with a new HIV diagnosis was highest among those aged 40–49 years (2.4% Medicaid, 1.4% commercial insurance) and lowest among males aged 13–14 years (0.3% Medicaid, 0.6% commercial insurance), p<0.0001 for both samples. The percentage of non-Hispanic blacks/African Americans with a new HIV diagnosis was higher (1.6%) compared with non-Hispanic whites (0.8%), Hispanics/Latinos (1.1%), and non-Hispanic males of other race/ethnicity (1.2%), p<0.0001 (Table 4).

Among male Medicaid patients, after adjusting for race/ethnicity, males aged 13–14 years (OR = 0.03, 95% CI 0.01, 0.12) and aged 15–19 years (OR = 0.5, 95% CI 0.3, 0.7) had lower odds of having HIV diagnosed compared with males aged 50–64 years. Males aged 40–49 years (OR = 1.8, 95% CI 1.3, 2.6) had higher odds. After adjusting for age, black/African Americans males (OR = 5.0, 95% CI 3.7, 6.8), Hispanic/Latino males (OR = 2.3, 95% CI 1.04, 5.04), and males of other race/ethnicity (OR = 2.2, 95% CI 1.4, 3.4), had higher odds of having HIV diagnosed compared with white males (data not shown).

### Additional Tests and Diagnoses during Outpatients Visits with HIV Testing

For females whose clinic visit included an HIV test, 74.7% of Medicaid and 72.0% of commercial insurance claims also included tests or diagnoses for another STI; these percentages increased to 81.6% and 84.2%, respectively, when hepatitis B or C tests or diagnoses were included (Table 5). Tests for syphilis were the most common, occurring in approximately two-thirds of visits with an HIV test for females with Medicaid or commercial insurance (Table 5).

**Table 5:** Tests and diagnoses for sexually transmitted infections, hepatitis B virus, and hepatitis C virus during visits with an HIV test among tests for females and tests for males, Medicaid and commercial insurance, 2012.

	Medicaid				Commercial insurance			
	Females		Males		Females		Males	
	(N = 77,424 visits)		(N = 22,991 visits)		(N = 474,081 visits)		(N = 335,456 visits)	
	N	%	N	%	N	%	N	%
Syphilis								
Test	49,556	64.0	11,729	51.0	316,280	66.7	198,286	59.1
Diagnosis	11	0.01	7	0.03	85	0.02	241	0.1
Any STI^a^ test or diagnosis	57,825	74.7	14,805	64.4	341,474	72.0	238,979	71.2
Hepatitis B virus								
Test	24,845	32.1	6,151	26.7	244,961	51.7	131,763	39.3
Diagnosis	69	0.1	45	0.2	364	0.1	451	0.1
Hepatitis C virus								
Test	23,449	30.3	6,501	28.3	241,701	51.0	141,467	42.2
Diagnosis	132	0.2	121	0.5	276	0.1	400	0.1
Any STI^a^, Hepatitis B or C	63,192	81.6	17,394	75.7	398,983	84.2	272,584	81.3
screening or diagnosis								

STI = sexually transmitted infection.

^a^Tests for gonorrhea or chlamydia during well visits among females aged 13 to 24 years were excluded.

For males whose clinical visit included an HIV test, 64.4% of Medicaid and 71.2% of commercial insurance claims also included tests or diagnoses for another STI; the percentage increased to 75.7% and 81.3%, respectively, when HBV or HCV testing or diagnoses were included. Tests for syphilis were the most common, occurring in 51.0% of visits with an HIV test for males with Medicaid and 59.1% for those with commercial insurance (Table 5).

## Discussion

Our analysis of claims data found that less than one in twenty patients with Medicaid and less than one in thirty patients with commercial insurance received a test for HIV during 2012. The vast majority of those tested for HIV in our study were also concurrently tested for other STIs, most notably syphilis, testing that is not routinely conducted in the absence of sexual risk factors or symptoms of infection. This finding suggests that most of the observed HIV testing was likely targeted based on risk behaviors or symptoms and not part of a routine HIV screening program. Nationally, less than 50% of persons report they have been tested for HIV, and the annual rates of HIV testing have remained stable since 2003 [6]. Since 2006, CDC and professional medical associations have recommended that all adolescents and adults (up to 65 years) be tested for HIV at least once. However, the USPSTF did not issue its recommendation until 2013. The USPSTF’s much later endorsement may be a significant factor for both the findings reported here and future HIV testing trends in the United States. Under provisions instituted by the Affordable Care Act (ACA), both HIV screening and targeted risk-based testing are now covered without cost-sharing as part of the essential benefits package available to all individuals enrolled in non-grandfathered private healthcare plans or Medicaid expansion plans (https://www.healthcare.gov/what-are-my-preventive-care-benefits/; accessed 9/19/2014). These policy changes have the potential to boost HIV testing rates, especially if coupled with supportive, multi-level implementation strategies (e.g., HIV testing awareness campaigns for patients, clinical decision support for providers, and population-based performance measure for health plans). Providers also need to be aware of national HIV testing guidelines and policies. Awareness of CDC’s 2006 recommendations for HIV screening has been low among primary care physicians, emergency department doctors, and internists [12, 13], but this might improve with the recommendation from the USPSTF.

One previous study assessed annual HIV testing using electronic medical record data [14]. That study, conducted among veterans receiving care through the Veterans Health Administration (VA), found a percentage of annual HIV testing at baseline (2.5%) similar to that in our commercial insurance sample (2.8%) [14]. After the VA issued a directive in August 2009 encouraging voluntary HIV testing at least once as part of routine medical care for all veterans, the percentage of annual HIV testing increased to 8.6%, and the cumulative percentage of veterans ever tested for HIV increased from 9.2% in 2009, before the directive, to 20.0% in 2011 [14]. Our estimates of HIV testing in 2012 among females and males are lower than self-reported recent HIV testing among non-pregnant persons aged 15–44 years in the 2006–2010 National Survey of Family Growth (NSFG) survey [15]. Among NSFG respondents covered through Medicaid, 44.1% of females (includes pregnant women) and 16.9% of males reported being tested for HIV in the last year; among those with commercial insurance, the corresponding figures were 18.5% for females (includes pregnant women) and 11.7% for males [15]. Discrepancies between health-care claims data and self-report of HIV testing in the preceding 12 months can be attributed to several factors. Self-reports include testing outside the health care system, such as at community events, in correctional facilities, and in other venues where targeted HIV testing takes place. In addition, differences in demographic and geographic characteristics of persons in the samples likely contributed to differences in the estimated percentage of persons tested for HIV in the last year. Although the percentages tested for HIV differed, the patterns by gender and age were similar among the NSFG, Medicaid, and commercial insurance samples. In addition, when comparing 2012 diagnoses rates in the United States to the findings in this study we found many consistencies [16]. Men had higher diagnoses rates than women, blacks had higher diagnoses rates than all other race/ethnicity groups, and persons aged 13–14 years had the lowest diagnoses rates [16]. The diagnoses rate in the other age groups were less consistent. Nationally, estimated rates of HIV diagnoses were highest among those aged 20–29 years, whereas we found higher diagnoses rates in older persons.

This study is subject to several limitations. Because we relied on administrative data tied to insurance claims, we may have overestimated the number of new HIV diagnoses, as persons tested and diagnosed with HIV may have previously received an HIV diagnosis (e.g., at a community testing event) before enrollment. On the other hand, we may have slightly underestimated diagnoses. In December 2012, 6,441 persons were tested for HIV in the Medicaid sample and 60,813 in the commercially-insured sample. Applying the positivity rate found during the entire year and subtracting the numbers diagnosed in December, we may have under-estimated the number of diagnoses by approximately 14 in the Medicaid sample and 80 in the commercially insured sample if the diagnoses were not recorded until January 2013.

Another limitation is that the commercial insurance sample is a convenience sample that Truven Health MarketScan purchased. Although this sample represents a significant proportion, approximately one-quarter, of commercial insurance claims in the United States, it may not be representative of all commercial insurance claims. In addition, because the states contributing to the Medicaid database were not identified, we could not assess the representativeness of these data for the broader Medicaid population. Finally, the findings of this study cannot necessarily be generalized to other populations such as those who are uninsured. The two samples in this study likely reflect very different populations. Persons eligible for Medicaid may have lower incomes and be less likely to be employed than persons with commercial insurance. Despite these differences, HIV testing was low in both samples. The number of persons with previously diagnosed HIV was proportionally higher among the Medicaid sample, while the number of persons with new diagnoses were only marginally higher. This may reflect the fact that a sizeable proportion of people living with diagnosed HIV infection qualify for disability benefits, and these benefits, in turn, also confer access to Medicare and Medicaid coverage [17]. Consequently, prior to the ACA, Medicaid was the single largest source of health insurance for people with HIV in the United States, and was estimated to cover a quarter of all people with diagnosed HIV [18].

### Conclusions

Our analysis of claims among persons insured through Medicaid and those insured through commercial insurance offers little evidence that routine HIV screening was being implemented during outpatient medical visits in 2012. Most HIV testing was provided during the same visit as testing or diagnosis of STIs, HBV, or HCV, suggesting that risk behaviors or symptoms prompted the HIV testing. The National HIV/AIDS Strategy has set a target for increasing the percentage of people living with HIV who are aware of their HIV infection to 90%. As of 2012, an estimated 87.2% of persons living with HIV were aware of their status [1]. The low percentages of persons tested for HIV in this study suggest that important opportunities to advance this national goal were missed and efforts are needed to increase routine screening for HIV in health care settings.
